# Factor structure of the Eating Disorder Examination-Questionnaire (EDE-Q) in adult men with eating disorders

**DOI:** 10.1186/s40337-023-00757-4

**Published:** 2023-03-06

**Authors:** Nora M. Laskowski, Georg Halbeisen, Karsten Braks, Thomas J. Huber, Georgios Paslakis

**Affiliations:** 1 University Clinic for Psychosomatic Medicine and Psychotherapy, Medical Faculty, Campus East-Westphalia, Ruhr-University Bochum, Virchowstr. 65, 32312 Luebbecke, Germany; 2 Centre for Eating Disorders, Klinik am Korso, 32545 Bad Oeynhausen, Germany

**Keywords:** Eating disorders, Eating Disorder Examination-Questionnaire, EDE-Q, Factor analysis, Men’s health, Body dissatisfaction, Muscularity

## Abstract

**Background:**

Previous investigations on the Eating Disorder Examination-Questionnaire (EDE-Q) factor structures in men have been restricted to non-clinical settings, limiting conclusions about the factorial validity in men with eating disorders (ED). This study aimed to examine the factor structure of the German EDE-Q in a clinical group of adult men with diagnosed ED.

**Methods:**

ED symptoms were assessed using the validated German version of the EDE-Q. Exploratory factor analysis (EFA) using principal-axis factoring based on polychoric correlations was conducted for the full sample (*N* = 188) using Varimax-Rotation with Kaiser-Normalization.

**Results:**

Horn’s parallel analysis suggested a five-factor solution with an explained variance of 68%. The EFA factors were labeled “Restraint” (items 1, 3–6), “Body Dissatisfaction” (items 25–28), “Weight Concern” (items 10–12, 20), “Preoccupation” (items 7 and 8), and “Importance” (items 22 and 23). Items 2, 9, 19, 21, and 24 were excluded due to low communalities.

**Conclusions:**

Factors associated with body concerns and body dissatisfaction in adult men with ED are not fully represented in the EDE-Q. This could be due to differences in body ideals in men, e.g., the underestimation of the role of concerns about musculature. Consequently, it may be useful to apply the 17-item five-factor structure of the EDE-Q presented here to adult men with diagnosed ED.

## Abstract (plainenglishlanguage)

Eating disorders (ED), such as Anorexia Nervosa, Bulimia Nervosa, and Binge-Eating Disorder, are more and more recognized as a public health concern for men, but men are still addressed insufficiently in ED research. Adequate assessment is essential for a deeper understanding of ED pathology in men. This is the first study to address the factor structure of the validated German version of the Eating Disorder Examination-Questionnaire (EDE-Q) in adult inpatient men with diagnosed ED by using exploratory factor analysis (EFA). The EFA yielded a five-factor structure with 17 items. The findings of this study offer novel insights into the applicability of the EDE-Q in adult men with ED.

## Background

Eating disorders (ED), such as Anorexia Nervosa (AN), Bulimia Nervosa (BN), and Binge-Eating Disorder (BED), are characterized by body image disturbances, abnormal eating patterns, and weight-control behaviors [1]. With an estimated lifetime prevalence of 2.6–8.4% in women and 0.7–2.2% in men, the public health relevance of ED is becoming increasingly relevant [2–4]. Consequences of ED are, for example, one of the leading risks of mortality among mental disorders [5] and enormous health and economic costs [6].

Although the perception that ED primarily affect women is still widely prevalent [7, 8], ED are also a significant health risk for men [9–11]. Since 1990, despite lower rates in general, the prevalence in men has increased faster than in women (in 2019 by 22% and 12% to 117.9 and 231.5 per 100 000 men and women, respectively; [12]). Similarly, disability-adjusted life years (DALYs) increased by 0.7% each year for men, compared to 0.6% for women [13]. Nevertheless, men’s representation in ED research remains lower than expected based on prevalence estimates [14], while experts agree that the available data may still underestimate the prevalence of men with ED, as men may be more reluctant to disclose their condition and seek treatment [15, 16].

Gender differences in ED pathology may play a central role in lowered recognition and underrepresentation of men in ED research, as standard assessments were developed based on ED presentation in adolescents and young women [17, 18]. Consequently, there have already been initial attempts to develop men-specific assessment instruments [19–21] as well as to validate existing ED assessments in men [22–24]. For example, the widely used Eating Disorder Examination-Questionnaire (EDE-Q; [25]), designed to capture the variation and severity in eating-related psychopathology [26], is based on common cognitive behavioral models of ED. Its items thus reflect the theory that the pursuit of thinness underlies ED [27]. Consistent with its conceptualization, the EDE-Q groups ED-associated pathology along four subscales labeled “Restraint”, “Eating Concern”, “Shape Concern”, and “Weight Concern” [25]. While the EDE-Q shows high levels of convergent validity, the validity of its factor structure remains an issue of ongoing discussion [28–30]. The original four-factor structure appears particularly problematic for men [23], for whom the concept of body image appears to be relevant, but a focus on the underlined “thinness ideal” is too limiting and may underestimate the role of concerns about musculature [27] -which, as we must note, are also increasingly relevant among women [31, 32]. These and similar findings question the validity of using the EDE-Q subscales for measuring ED pathology in men. Indeed, several studies have shown difficulties reproducing the original EDE-Q factor structure, including in cohorts of men [24, 33]. This indicates that the original factor model may not be sensitive to different ED manifestations in adult men [23, 24]. For example, Klimek et al. [34] showed that in men who identify as gay, bisexual, or otherwise non-heterosexual (*n* = 479), the original four-factor solution could not be applied, similar to a male Argentine sample consisting of university students and athletes (*n* = 986; [35]).

Thus far, however, previous investigations on EDE-Q factor structures in men have been limited to non-clinical settings [36, 37], severely limiting conclusions about the EDE-Q’s factorial validity among men with diagnosed ED. For example, it is currently unknown whether and which subscale scores should be reported for men with ED, or which subscale could be used to compare men with ED to other samples, or for tracking treatment response [24]. Hence, analysis and, if necessary, adaptation of the factor structure to specific patient groups are elementary for valid ED assessment. Therefore, the purpose of the current study was to explore the factor structure of the German EDE-Q in a clinical group of adult men with ED.

## Methods

### Participants and measures

The participants in this study were adult men with ED admitted for inpatient treatment (*N* = 188, consisted of a subsample of previously described patients) [10]. Thus, data were retrospectively analyzed. The patients had been registered for inpatient ED-specific treatment at the Klinik am Korso, Bad Oeynhausen, Germany, between January 2018 and December 2021, and received consecutive treatment at least once. Diagnoses according to ICD-10 criteria [38] were based on the clinical judgment of long-term experts in the diagnosis and treatment of ED and validated by means of peer review (group meetings). For one man with AN treated more than once at the clinic, only the data from the first admission were selected for analysis. Exclusion criteria were absence of an ED and gender other than male. In addition, only adult patients were included. The study had been approved under application AZ 2021–849 by the Ethics Committee of the Medical Faculty of the Ruhr-University Bochum at the East Westphalia Campus. The study was also registered prospectively in the German Clinical Trials Registry as part of application DRKS00028441. Data sets and analysis scripts are available from the authors upon request.

Basic data (demographics, height, and weight) of patients were recorded at admission. Height and body weight were used to compute body mass index (BMI). General psychopathology was assessed with the Symptom Checklist-27-plus (SCL-27-plus; [39]), a modification of the Symptom Checklist-90-R (SCL-90-R; [40]) with 28 items in five subscales (“Depressive Symptoms”, “Vegetative Symptoms”, “Agoraphobic Symptoms”, “Sociophobic Symptoms” and “Pain”). The answering format ranged on a five-point scale from 0 (never) to 4 (very often). Additionally, the widely used Beck Depression Inventory (BDI-I; [41]) was used to assess depression symptom severity with 21 items. The answering format ranged on a five-point scale from 0 to 4, the sum score ranged between 0 and 63. Scores up to 9 indicate minimal depression, scores of 10–18 mild depression, scores of 19–29 moderate depression, and scores of 30 and above indicate severe depression.

The cognitive and behavioral symptoms of ED were assessed using the EDE-Q. Due to the retrospective nature of the analyses, no other men-specific aspects (e.g., measures of muscularity) were available. The EDE-Q [25] is a self-assessment questionnaire assessing core ED symptoms over the past 28 days. It is based on the Eating Disorder Examination Interview [42], includes definitions and time frames for major symptoms, and can usually be completed in minutes. Here, the validated German translation [43, 44] which contains 28 items, was used. Each item was rated on a seven-point scale ranging between 1 (never) and 7 (every day). For comparability with the original coding, all data were recoded from 0 to 6 for analysis. The EDE-Q is evaluated using sum values. There is a total sum value next to sum values for four subscales that are usually determined (“Restraint”, “Eating Concern”, “Shape Concern”, and “Weight Concern”; [45]). Six further open questions assess the frequencies of compensatory behaviors and objective binge-eating. These are generally not included in the evaluation and calculation of the subscales and were also excluded from the analysis in the present context (items nos. 13 to 18). The internal consistency of the subscales of the EDE-Q was examined in four studies and showed acceptable values ranging from 0.70 to 0.93 (examined in female students and treatment-seeking men and women with BED). Re-test reliability data ranged from *r* = 0.66 to *r* = 0.94 (examined in female students, in female community samples, and in women with BN [26]). Individuals are usually classified into the clinical range based on a cut-off score of ≥ 4 (on one of the four subscales and/or total score) of the EDE-Q. The EDE-Q was applied in a standardized manner at the beginning and end of treatment. Only admission data were used for the following analyses; we did not consider using the EDE-Q data collected post-treatment for factorial validation given that treatment effects may interfere with the applicability of many of the items.

### Statistical analyses

Because of the inconsistency of the internal structure of the EDE-Q across different samples as described above, it did not seem reasonable to assume that the structure of the EDE-Q found in other studies is the same in adult inpatient men with ED. Therefore, an exploratory—rather than a confirmatory—approach was chosen to analyze the factor structure of the German EDE-Q. All available data were included (missing data < 5%, data were missing completely at random according to Little's missing completely at random [MCAR] test, *χ*^*2*^ (df = 120) = 120,* p* = 0.48). The Kaiser–Meyer–Olkin measure of sampling adequacy (KMO) was used to ensure the conductibility of the exploratory factor analysis (EFA) as recommended by Dziuban and Shirkey [46]. A result near 1.0 on the KMO indicated the amount of variance of variables that could be explained by underlying factors (a minimum value of 0.5 is required; [46]). Because of their increased robustness against normality violations, EFA of polychoric correlations using principal-axis factoring was used [47]. Varimax-Rotation with Kaiser-Normalization was chosen for factor rotations to maximize the variance of the squared loadings across a factor, rather than the variance of the squared loadings for the variables [48]. Horn’s parallel analysis is considered among the best empirical methods for factor retention in factor analysis [49, 50] and was used to determine the number of factors to retain. Based on the idea that real data with an underlying factor structure should produce factors with eigenvalues larger than those derived from random data sets with identical numbers of cases and variables [51], parallel analysis compares, from first to last extracted, eigenvalues from real data with average eigenvalues from a large number of constructed random data sets (5 000, in our case). A factor is retained if its eigenvalue is larger than the parallel average eigenvalue derived from the random data sets (i.e., the adjusted difference between real and random data eigenvalues > 0). Item retainment was based on the results of an initial EFA based on the communalities after extraction, which needed to be higher than 0.3. Items with high factor loadings defined each factor and each item was referred to the factor in which it had the highest loading. Each item had to have a (moderate) loading over 0.40, as to be a clinically meaningful item in one of these factors and based on standard criteria [52]. If this value was not met, the affected items were excluded and the analysis was repeated. In addition, we considered item cross-loadings, with relative load differences below 0.20 defined as the threshold [48, 53], although we ultimately based dropping or retaining cross-loading items on their theoretical plausibility and their implications for factor structure.

For data analyses, we used SPSS Statistics version 28 for Windows [54] and R version 4.2.2 [55], using packages naniar 1.0.0 for MCAR test [56], EFA.dimensions 0.1.7.6 [57] for calculating polychoric correlations (using method “Fox”) and psych 2.2.9 [58] for the feasibility test, parallel analysis, and the EFA. Figure 2 is based on R package DandEFA 1.6 [59]. Descriptive statistics (means and SD) were computed for demographic characteristics and factors. In addition, Spearman correlations were calculated for the factors, for the factors and the BMI, for the factors and the BDI-I, and the factors and the SCL-27-plus subscales (“Depressive Symptoms”, “Vegetative Symptoms”, “Agoraphobic Symptoms”, “Sociophobic Symptoms” and “Pain”). For reliability analysis, Cronbach’s alpha was calculated to assess the internal consistency of the factors; values of 0.8 and above were considered good. As this is a retrospective analysis, no power planning had been carried out in advance.

## Results

### Sample characteristics

The characteristics of the sample are shown in Table 1.

**Table 1:** Sample Descriptive Statistics

Variable	n	M	SD
Age	188	32.5	13.1
Body mass index	188	37.8	15.5
Eating Disorder Examination-Questionnaire	188	3.37	1.1
Beck Depression Inventory I	186	24.0	11.8
Symptom Checklist-27-plus: Depressive Symptoms	186	1.9	1.0
Symptom Checklist-27-plus: Vegetative Symptoms	186	1.4	0.8
Symptom Checklist-27-plus: Agoraphobic Symptoms	186	1.0	0.9
Symptom Checklist-27-plus: Sociophobic Symptoms	186	2.1	1.1
Symptom Checklist-27-plus: Pain	186	1.8	0.8
*Diagnosis*			
Anorexia Nervosa	44		
Bulimia Nervosa	18		
Binge-Eating Disorder	116		
Eating Disorder not otherwise specified	10		

Bold type highlights the respective loadings of the items that were crucial for the assignment to the respective factors

### Feasibility tests for exploratory factor analysis

Because of their increased robustness against normality violations, EFA of polychoric correlations using principal-axis factoring was used [47, 60]. The overall KMO (0.80) confirmed that the polychoric correlation matrix of items was factorable.

### Results of the exploratory factor analysis

**Fig. 1:**
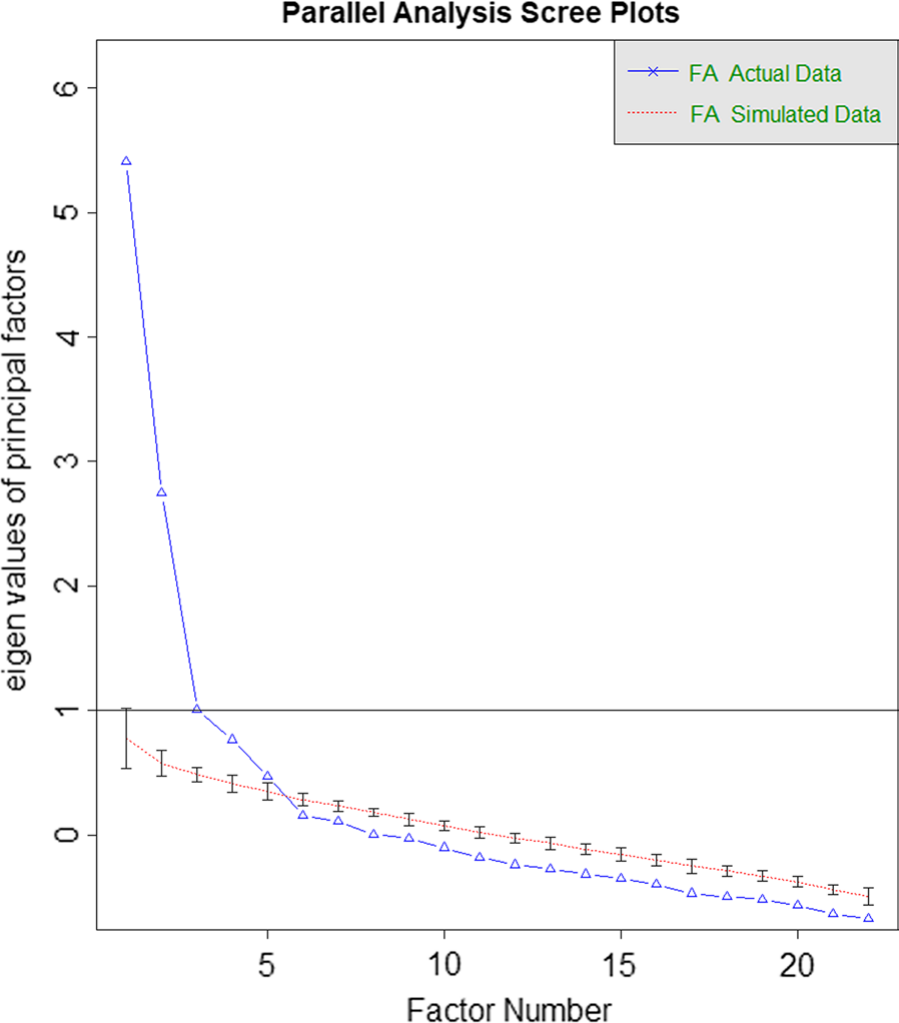
Horn’s parallel analysis

Horn’s parallel analysis [49, 50, 61] showed five factors with adjusted eigenvalues greater than 0, suggesting that five factors should be retained (see Fig. 1). The initial EFA revealed four items with communality values below 0.3 after extraction (nos. 2, 19, 21, 24), and one item with loading less than 0.4 (-0.05—0.37) across factors (no. 9). These items were therefore excluded. The second and final EFA included 17 of the original 22 items, which all met minimal communality and loading criteria. Two items (nos. 10 and 20) presented with cross-loadings based on relative loading difference criteria. However, because the cross-loadings were theoretically plausible (see below) and excluding the items neither reduced the number of suggested factors in parallel analysis nor affected the other items’ loading structure, we retained both items.

Table 2 presents the communality and loading of the items across factors. Items 1 and 3–6 primarily loaded on factor 1 (eigenvalue [EV] = 3.03, variance explained [VE] = 0.26), items 25–28 on factor 2 (EV = 2.80, VE = 0.24), items 10–12 and 20 on factor 3 (EV = 2.06, VE = 0.18), items 7 and 8 on factor 4 (EV = 1.90, VE = 0.17), and items 22 and 23 on factor 5 (EV = 1.69, VE = 0.15). All loadings ranged from 0.45 to 0.86. Thirteen of the factor loadings were above 0.70, which corresponded to an ideal loading [52], and all had values of more than 0.40, that according to Swami and Barron, [47] the sample size may be considered to be suitable.

**Table 2:** Item loadings on factors

Item		Communality	Factor 1: Restraint	Factor 2: Body Dissatisfaction	Factor 3: Weight Concern	Factor 4: Preoccupation	Factor 5: Importance
1	Restraint over eating ^R^	0.62	**0.77**	−0.06	0.12	0.00	0.14
2	Avoidance of eating ^R^	(0.29) *	item excluded ^a^				
3	Food avoidance ^R^	0.67	**0.78**	−0.09	0.08	0.14	0.16
4	Dietary rules ^R^	0.57	**0.73**	−0.04	0.05	0.14	0.05
5	Empty stomach ^R^	0.59	**0.70**	0.07	0.01	0.31	-0.06
6	Flat stomach ^SC^	0.45	**0.57**	0.11	0.10	0.31	-0.09
7	Preoccupation with food, eating or calories ^EC^	0.79	0.33	−0.09	0.02	**0.81**	0.14
8	Preoccupation with shape or weight ^WC, SC^	0.77	0.27	0.04	0.10	**0.83**	0.09
9	Fear of losing control over eating ^EC^	(0.39) *	item excluded ^b^				
10	Fear of weight gain ^SC^	0.64	0.46	0.18	**0.54**	0.333	0.09
11	Feelings of fatness ^SC^	0.75	0.05	0.36	**0.77**	0.03	0.14
12	Desire to lose weight ^WC^	0.80	0.08	0.25	**0.85**	0.00	0.11
19	Eating in secret ^EC^	(0.16) *	item excluded ^a^				
20	Guilt about eating ^EC^	0.49	0.21	0.27	**0.45**	0.39	0.14
21	Social eating ^EC^	(0.29) *	item excluded ^a^				
22	Importance of weight ^WC^	0.87	0.10	0.33	0.17	0.11	**0.84**
23	Importance of shape ^SC^	0.90	0.11	0.33	0.15	0.14	**0.86**
24	Reaction to prescribed weighing ^WC^	(0.18) *	item excluded ^a^				
25	Dissatisfaction with weight ^WC^	0.70	−0.12	**0.76**	0.25	−0.11	0.15
26	Dissatisfaction with shape ^SC^	0.80	−0.01	**0.86**	0.21	−0.04	0.15
27	Discomfort seeing body ^SC^	0.65	0.07	**0.77**	0.17	0.01	0.17
28	Avoidance of exposure ^SC^	0.41	−0.03	**0.58**	0.14	0.18	0.17
Variance explained			18%	16%	12%	11%	10%

Bold type highlights the respective loadings of the items that were crucial for the assignment to the respective factors

Rotation method: Varimax-Rotation with Kaiser-Normalization

^R ^= Restraint; ^EC^ = Eating Concern; ^WC^ = Weight Concern; ^SC^ = Shape Concern; Indexed abbreviations indicate item assignment to one of the four original EDE-Q subscales (R, EC, WC, SC)

*The communalities of the excluded items are from the first EFA with all 22 items

^a^These items were excluded since communality was lower than 0.30

^b^This item was excluded since all factor loadings were below 0.40

The factors explained 68% of the total variance, which is to be considered a high percentage. Cronbach’s α indicated high internal consistencies across factors 1 to 5 (i.e., 0.85, 0.90, 0.83, 0.89, and 0.95, respectively), suggesting adequacy of the five-factor solution.

Spearman correlations calculated for the factors, for the factors and the BMI, for the factors and the BDI, and the factors and the SCL-27-plus subscales (“Depressive Symptoms”, “Vegetative Symptoms”, “Agoraphobic Symptoms”, “Sociophobic Symptoms” and “Pain”) are given in Table 3.

**Table 3:** Spearman-Correlations of the EDE-Q subscale means, the SCL-27-plus, the BDI, and the BMI

	Factor					SCL-27-plus					BDI-I	BMI
	1	2	3	4	5	DS	VS	AS	SS	Pain		
*Factor*												
1	–	.06 [−0.09; 0.20]	.43 [0.31; 0.55]	.51 [0.39; 0.61]	.21 [0.07; 0.35]	.25 [0.10; 0.38]	.15 [0.00; 0.29]	.24 [0.09; 0.37]	.32 [0.18; 0.44]	.02 [−0.13; 0.17]	.32 [0.19; 0.45]	−.32 [−0.45; −0.19]
2		–	.44 [0.31; 0.55]	.11 [−0.04; 0.25]	.51 [0.39; 0.61]	.21 [0.07; 0.35]	.11 [−0.04; 0.25]	.22 [0.07; 0.35]	.34 [0.21; 0.47]	.27 [0.13; 0.41]	.28 [0.14; 0.41]	.34 [0.20; 0.46]
3			–	.42 [0.29; 0.54]	.46 [0.33; 0.56]	.26 [0.12; 0.40]	.20 [0.05; 0.33]	.24 [0.10; 0.38]	.29 [0.15; 0.42]	.22 [0.07; 0.35]	.33 [0.19; 0.45]	.22 [0.07; 0.35]
4				–	.26 [0.12; 0.39]	.38 [0.24; 0.50]	.21 [0.07; 0.35]	.22 [0.07; 0.35]	.28 [0.13; 0.41]	.03 [−0.12; 0.18]	.40 [0.26; 0.51]	−.22 [−0.36; −0.08]
5					–	.27 [0.13; 0.40]	.21 [0.06; 0.40]	.31 [0.17; 0.44]	.40 [0.26; 0.51]	.21 [0.07; 0.35]	.39 [0.25; 0.51]	.18 [0.04; 0.32]
*SCL-27-plus*												
DS						–	.55 [0.43; 0.64]	.46 [0.33; 0.57]	.51 [0.39; 0.61]	.29 [0.15; 0.42]	.75 [0.68; 0.81]	−.13 [−0.28; 0.01]
VS							–	.56 [0.45; 0.65]	.44 [0.31; 0.55]	.49 [0.37; 0.59]	.48 [0.36]	−.18 [−0.32; −0.03]
AS								–	.49 [0.37; 0.60]	.35 [0.22; 0.48]	.51 [0.40; 0.62]	−.09 [−0.23; 0.06]
SS									–	.33 [0.19; 0.46]	.57 [0.46; 0.66]	−.07 [−0.21; 0.08]
Pain										–	.32 [0.18; 0.45]	.19 [0.04; 0.33]
BDI											–	−0.09 [−0.24; 0.05]
BMI												–

[95% confidence intervals]; DS = Depressive Symptoms; VS = Vegetative Symptoms; AS = Agoraphobic Symptoms; SS = Sociophobic Symptoms; BDI-I = Beck Depression Inventory I; BMI = body mass index

### Content evaluation of the calculated factor structure

**Fig. 2:**
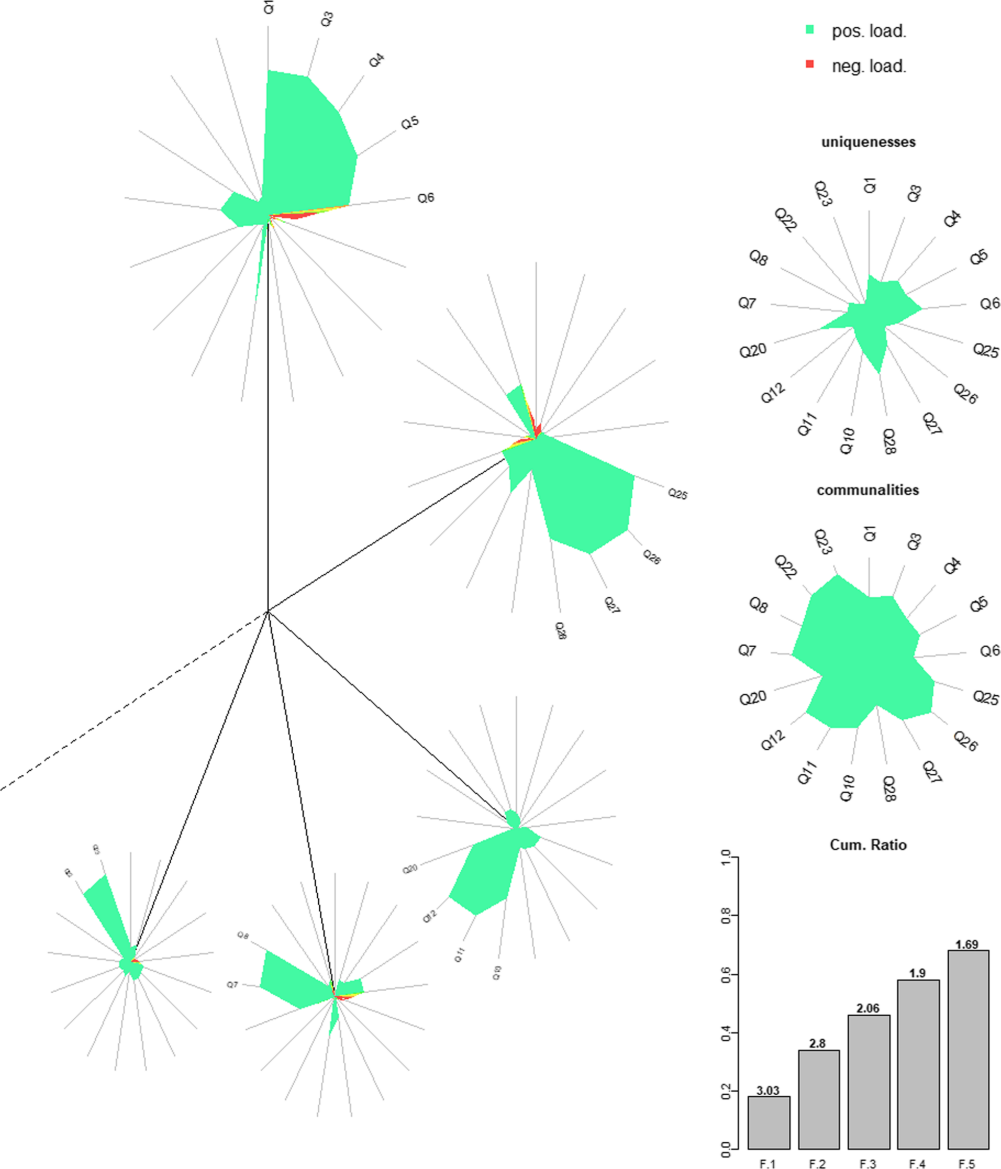
Dandelion plot of rotated factor solution [59]. *Note.* Central lines represent different factors. Star graphs visualize item factor loadings for each factor, with negative and positive loadings indicated by red and green, respectively. The size of each star graph and angles between solid lines represent the proportion of variance explained by each factor. The dashed line (starting from the solid line at 12 o'clock) indicates the cumulative percent of variance explained by the factor solution. Communalities and uniquenesses are shown on the right-hand side along with a bar chart of cumulative explanation ratios of factors (with individual factor variances [EV] on top)

Labeling of the factors was based on a content assessment of the items with primary loadings, which were assigned to different constructs in their entirety. Compared to the item assignment of the original subscales, the “Restraint” subscale was not substantially different in the present study (factor 1). Differences were that one item from the original subscale was removed (item no 2), and item no 6 (“Flat stomach”) was added. As shown in Fig. 2, factor 2, labeled “Body Dissatisfaction”, included dissatisfaction with weight and figure, and discomfort as well as avoidance of exposure, representing a subset of the original “Weight Concern” and “Shape Concern” items. Factor 3, labeled “Weight Concern”, contained four “Eating, Weight, and Shape Concern” items related to weight loss. Finally, Factors 4 and 5 emerged as two-item solutions, independently representing “Preoccupation” (with food/calories, shape/weight) items and “Importance” (of shape and weight) items across the original “Concerns” subscale. Similar to the original EDE-Q, two items, both from the “Weight Concern” scale, presented with cross-loadings: Item 10 (“Fear of weight gain”) also loaded on “Restraint”, and item 20 (“Guilt about eating”) also loaded on “Preoccupation”. However, because the fear of gaining weight could be reasonably construed as motivating “Restraint”, and “Preoccupation” might co-occur with the feeling of guilt, we decided to retain both items, although future reformulation may be advised.

**Fig. 3:**
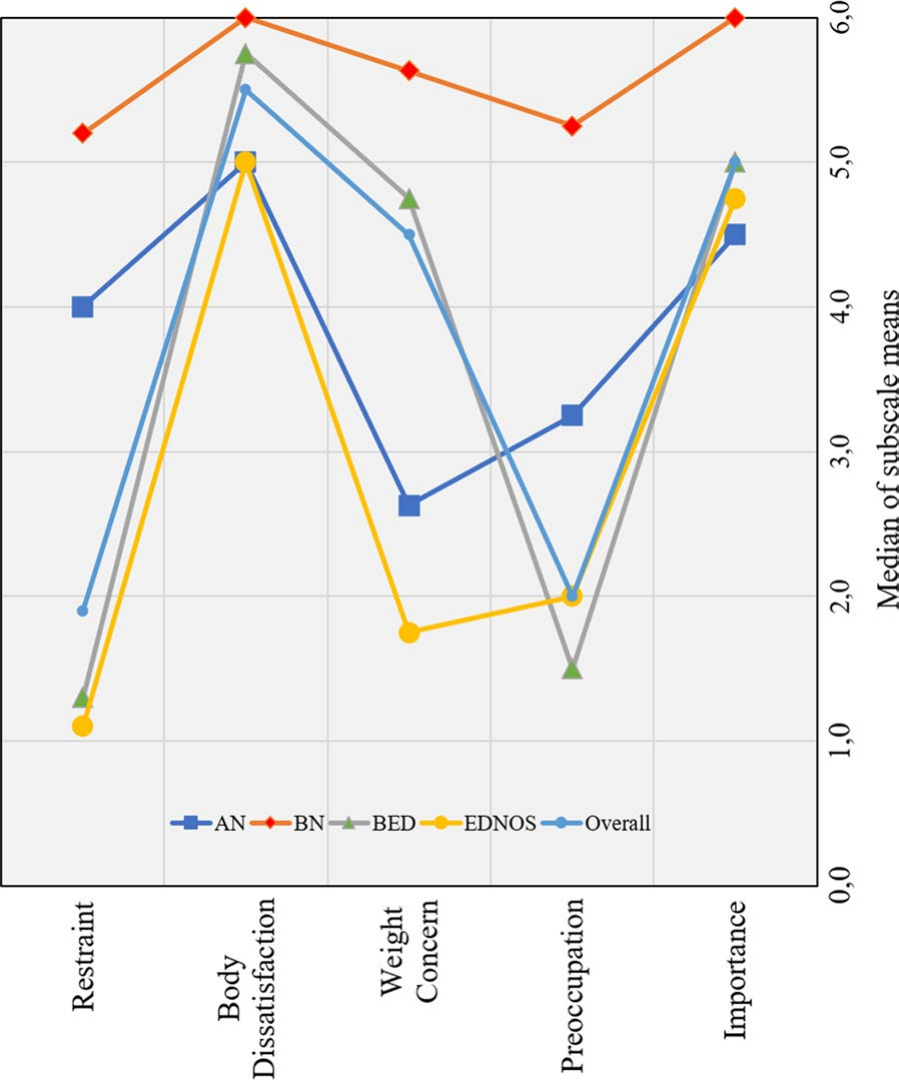
Patient group profiles across proposed EDE-Q factors

For further exploration, we examined patient group profiles across EDE-Q factors (Fig. 3). “Restraint” (factor 1) appears to separate patients with AN or BN from patients with BED or EDNOS. “Weight Concern” (factor 3) was most pronounced for patients with BN, followed by patients with BED, AN, and EDNOS. “Preoccupation” (factor 4) was most pronounced for patients with BN, with the remaining groups all scoring similarly, but lower than in BN in comparison. All patient groups exhibited high—and similar—scores on “Body Dissatisfaction” (factor 2) and “Importance” (factor 5).

## Discussion

ED are increasingly recognized as a public health risk for men [12], but the inclusion of men in ED research remains low [14]. To address this issue, initial efforts are underway to validate existing ED assessments in men, such as with the EDE-Q [22, 23]. However, previous research on the factor structure of the EDE-Q in men has been limited to non-clinical settings, which severely limits conclusions about the factorial validity of the EDE-Q in adult men diagnosed with ED. In the current study, the factor structure of the German EDE-Q was investigated in a clinical group of adult men with ED.

For the present sample, we propose a five-factor solution with 17 items, after excluding several items due to low communality or insufficient factor loadings in the EFA (see also [62, 63]). Although the EDE-Qs primary purpose resides in capturing variation in ED psychopathology rather than in distinguishing diagnostic groups, we obtained a first factor that represents “Restraint”, similar to the original EDE-Q and other studies with men [28, 37, 62–65], that appears to separate patients with AN or BN from patients with BED or EDNOS. “Weight Concern” (factor 3) appeared to primarily distinguish patients with binge-eating behavior (i.e., patients with BN or BED) from the other diagnostic groups, and “Preoccupation” (factor 4), describing subjective impairments due to weight and shape concerns, appeared to further separate patients with BN from other groups. Across diagnostic groups, however, all patients reported high levels on two emerging factors, “Body Dissatisfaction” (factor 2) and “Importance” (factor 5), suggesting these factors might mark core ED symptoms that may distinguish patients from non-patient populations.

Is a five-factor model of the EDE-Q justified? Other studies with non-clinical samples of men proposed four, three, two, or even one-factor solutions [37], raising the question if more parsimonious proposals could be appropriate. However, we believe that maintaining a distinction between “Weight Concern”, “Body Dissatisfaction”, and “Importance” is justified in a clinical sample of men, given the fact that the EDE-Q was primarily designed to assess body concerns in women. The EDE-Q includes items that assess general (e.g., “Discomfort seeing body”) or more thinness-related dissatisfaction (e.g., “Discomfort with shape”). Thus, one might therefore reasonably argue that the independent emergence of “Body Dissatisfaction”, “Weight Concern”, and (likely thinness-related) “Importance” might mirror the fact that a “thinness ideal” is too limiting to capture ED-related psychopathology in men, and that a sizeable group of men in our sample may have been dissatisfied with their body, not due to the fear of weight gain or the self-concept centrality of thinness, but for example due to dissatisfaction with their body mass and musculature [27]. Of course, many women may be similarly dissatisfied with their body mass and musculature [31, 32], highlighting the general need to consider a broad range of body image phenotypes in ED psychopathology assessments. Whether or not the distinction between Body Dissatisfaction, Weight Concern, and Importance can be maintained while including muscularity-focused items is to be examined in future investigations; for the time being, the suggested distinction may provide a useful heuristic for exploring additional men-related body image concerns in clinical samples. Future studies may also use weighting algorithms (i.e., the weighting of specific items depending on the cohort under investigation) and/or specific norms of the EDE-Q to capture different ED phenotypes among men, although we should ultimately strive towards the development of gender-inclusive diagnostic tools.

Can we generalize the proposed five-factor model beyond German men with ED? Our findings reinforce the apparent lack of invariance of the EDE-Q factor structure across different samples, which has remained a source of ongoing controversy and investigation [24, 36]. The EDE-Q items appear to group differently across different populations, and we further observed a tendency for several items to cross-load on multiple factors, which may hint at an instability of factor structures even within defined populations. In fact, we retained items rather liberally out of clinical considerations despite presenting with cross-loadings, which is not an ideal situation in psychometric evaluation. We speculate that the presence of cross-loadings may reflect an issue (or, depending on one’s perspective, a feature) of the EDE-Qs item construction, which often seems to query a theoretical conjunction of ED psychopathology and behaviors. However, there may be mutually non-exclusive causes of restraint eating or body image dissatisfaction other than thinness concerns, and the lack of population invariance of the EDE-Q and the cross-loading of items might thus simply reflect that individuals interpret and answer these conjoined items differently. In this sense, the current model, while not assuming universal validity, might help to inform the construction and extension of revised measurement items.

In discussing the present findings, it is important to note several potential limitations. Although we are not aware of any systematic comparisons of the EDE-Q factor structure based on sample characteristics (like gender, age, or treatment setting), we cannot exclude that specific sociodemographic variables might, at least in part, account for the observed structural disparities. In a similar vein, we must note that factor analysis maximizes variance explanation based on sample properties, which would lead to a lower variance explanation of the same factor solution given a different sample (i.e., “shrinkage”, see [66]). Similarly, we must caution against a definitive interpretation of the “Preoccupation” and “Importance” factors, since the obtained two-item solutions are under-identified (i.e., there are fewer unique variance and co-variance estimates than parameters in the measurement models) and would thus be difficult to validate [67]. Ideally, at least three items per factor should be included for factorial validation, suggesting a need for extending the range of items in similar future investigations. Further validation of the identified structure is therefore needed.

We further note that our sample may be considered of marginal size for conducting an EFA. Moreover, as this was a retrospective analysis, there were no further questionnaire data available (e.g., measures of muscularity), which should be included in future research to test for convergent validity. In a similar vein, we were unable to compare the patients’ responses and evaluate their ED severity using reference data, as norms for the German EDE-Q are thus far only available for general population samples [68]. The data were collected exclusively at an ED-specific clinic, which has the benefit of reducing variation among different treatment settings and other ecological determinants, while also limiting generalizability and transferability to other samples due to promoting symptom homogeneity. Another limitation is that although the diagnoses were made based on an expert clinical interview, they were not based on a standardized structured interview (such as the EDE interview). We also presented data aggregated across different ED diagnoses, limiting conclusions about the presence or absence of differences between factor structures among different diagnostic groups. Exploring these differences and commonalities ideally requires larger patient samples (> 150 per diagnostic group) and should be pursued to determine the EDE-Q factorial validity among different diagnostic groups. Further explorations may also consider including EDE-Q frequency items in addition to attitudinal items. Similar to other investigations [24, 33], we only included attitudinal items for use in EFA while excluding open-ended frequency items, although reformulation and mutual integration are viable [69]. Last but not least, because of the displayed instability in the factor structure in specific cohorts, it may be worth to investigate the diagnosis-specific EDE-Q factor structure in women (AN vs. BN. vs. BED).

## Conclusions

In summary, we analyzed and presented a proposal for a factor structure of the German EDE-Q in adult inpatient men with ED. With 17 items of the original version included and the five subscales being “Restraint”, “Body Dissatisfaction”, “Weigh Concern”, “Preoccupation”, and “Importance”, the pursuit of a muscular body ideal in men may not be adequately assessed in the EDE-Q in its present form. These results add to the overall limited evidence regarding diagnostic tools available for men and may promote the development of future adequate measures.

## Data Availability

The datasets used and/or analyzed during the current study are available from the corresponding author upon reasonable request.

## Abbreviations

EDE-Q: Eating Disorder Examination-Questionnaire
ED: Eating disorders
EFA: Exploratory factor analysis
AN: Anorexia Nervosa
BN: Bulimia Nervosa
BED: Binge-Eating Disorder
DALYs: Disability-adjusted life years
BMI: Body mass index
SCL-27-plus: Symptom Checklist-27-plus
SCL-90-R: Symptom Checklist-90-R
BDI-I: Beck Depression Inventory
KMO: Kaiser–Meyer–Olkin measure of sampling adequacy
EV: Eigenvalue
VE: Variance explained
EDNOS: Eating Disorder not otherwise specified

## References

[1] American Psychiatric Association. DSM-5 Table of Contents - Section I: DSM-5 Basics; 2013.

[2] Galmiche M, Déchelotte P, Lambert G, Tavolacci MP (2019). Prevalence of eating disorders over the 2000–2018 period: a systematic literature review. Am J Clin Nutr.

[3] Qian J, Wu Y, Liu F, Zhu Y, Jin H, Zhang H (2022). An update on the prevalence of eating disorders in the general population: a systematic review and meta-analysis. Eat Weight Disord.

[4] Santomauro DF, Melen S, Mitchison D, Vos T, Whiteford H, Ferrari AJ (2021). The hidden burden of eating disorders: an extension of estimates from the Global Burden of Disease Study 2019. Lancet Psychiatry.

[5] Chesney E, Goodwin GM, Fazel S (2014). Risks of all-cause and suicide mortality in mental disorders: a meta-review. World Psychiatry.

[6] Streatfeild J, Hickson J, Austin SB, Hutcheson R, Kandel JS, Lampert JG (2021). Social and economic cost of eating disorders in the United States: evidence to inform policy action. Int J Eat Disord.

[7] Douglas V, Balas B, Gordon K (2021). Facial femininity and perceptions of eating disorders: a reverse-correlation study. PLoS ONE.

[8] Gordon KH, Perez M, Joiner TE (2002). The impact of racial stereotypes on eating disorder recognition. Int J Eat Disord.

[9] Gorrell S, Murray SB (2019). Eating disorders in males. Child Adolesc Psychiatr Clin N Am.

[10] Halbeisen G, Brandt G, Paslakis G (2022). A plea for diversity in eating disorders research. Front Psychiatry.

[11] Richardson C, Paslakis G (2021). Men’s experiences of eating disorder treatment: a qualitative systematic review of men-only studies. J Psychiatr Ment Health Nurs.

[12] Ferrari A (2022). Global, regional, and national burden of 12 mental disorders in 204 countries and territories, 1990–2019: a systematic analysis for the Global Burden of Disease Study 2019. Lancet Psychiatry.

[13] Wu J, Liu J, Li S, Ma H, Wang Y (2020). Trends in the prevalence and disability-adjusted life years of eating disorders from 1990 to 2017: Results from the Global Burden of Disease Study 2017. Epidemiol Psychiatr Sci.

[14] Flores LE, Muir R, Weeks I, Murray HB, Silver JK (2022). Analysis of age, race, ethnicity, and sex of participants in clinical trials focused on eating disorders. JAMA Netw Open.

[15] Weltzin TE, Cornella-Carlson T, Fitzpatrick ME, Kennington B, Bean P, Jefferies C (2012). Treatment issues and outcomes for males with eating disorders. Eat Disord.

[16] Murray SB, Nagata JM, Griffiths S, Calzo JP, Brown TA, Mitchison D (2017). The enigma of male eating disorders: a critical review and synthesis. Clin Psychol Rev.

[17] Forbush KT, Wildes JE, Pollack LO, Dunbar D, Luo J, Patterson K (2013). Development and validation of the Eating Pathology Symptoms Inventory (EPSI). Psychol Assess.

[18] Mitchison D, Mond J (2015). Epidemiology of eating disorders, eating disordered behaviour, and body image disturbance in males: a narrative review. J Eat Disord.

[19] Murray SB, Brown TA, Blashill AJ, Compte EJ, Lavender JM, Mitchison D (2019). The development and validation of the muscularity-oriented eating test: a novel measure of muscularity-oriented disordered eating. Int J Eat Disord.

[20] Tylka TL, Bergeron D, Schwartz JP (2005). Development and psychometric evaluation of the Male Body Attitudes Scale (MBAS). Body Image.

[21] Hildebrandt T, Walker DC, Alfano L, Delinsky S, Bannon K (2010). Development and validation of a male specific body checking questionnaire. Int J Eat Disord.

[22] Rica R, Solar M, Compte EJ, Sepúlveda AR (2022). Establishing the optimal male cut-off point: confirmatory factor analysis of the Eating Disorder Examination-Questionnaire (EDE-Q) in a representative sample of Spanish university students. Eat Weight Disord.

[23] Schaefer LM, Smith KE, Leonard R, Wetterneck C, Smith B, Farrell N (2018). Identifying a male clinical cutoff on the Eating Disorder Examination-Questionnaire (EDE-Q). Int J Eat Disord.

[24] Jenkins PE, Rienecke RD (2022). Structural validity of the Eating Disorder Examination—Questionnaire: a systematic review. Int J Eat Disord.

[25] Fairburn CG, Beglin SJ, Fairburn CG (2008). Eating Disorder Examination Questionnaire (EDE-Q 6.0). Cognitive behaviour therapy and eating disorders.

[26] Berg KC, Peterson CB, Frazier P, Crow SJ (2012). Psychometric evaluation of the eating disorder examination and eating disorder examination-questionnaire: a systematic review of the literature. Int J Eat Disord.

[27] Forrest LN, Perkins NM, Lavender JM, Smith AR (2019). Using network analysis to identify central eating disorder symptoms among men. Int J Eat Disord.

[28] Carey M, Kupeli N, Knight R, Troop NA, Jenkinson PM, Preston C (2019). Eating disorder examination questionnaire (EDE-Q): Norms and psychometric properties in U.K. females and males. Psychol Assess..

[29] Bardone-Cone AM, Boyd CA (2007). Psychometric properties of eating disorder instruments in Black and White young women: internal consistency, temporal stability, and validity. Psychol Assess.

[30] Black CMD, Wilson GT (1996). Assessment of eating disorders: interview versus questionnaire. Int J Eat Disord.

[31] Steinfeld B, Hartmann A, Waldorf M, Vocks S (2020). Development and initial psychometric evaluation of the body image matrix of thinness and muscularity—female bodies. J Eat Disord.

[32] Wilhelm L, Hartmann A, Becker J, Waldorf M, Vocks S (2020). Are there associations between religious affiliation and drive for muscularity? A cross-sectional survey of young Muslim women, Christian women and atheist women from Germany. BMC Womens Health.

[33] Grilo CM, Henderson KE, Bell RL, Crosby RD (2013). Eating disorder examination-questionnaire factor structure and construct validity in bariatric surgery candidates. Obes Surg.

[34] Klimek P, Convertino AD, Pennesi JL, Gonzales M, Roesch SC, Nagata JM (2021). Confirmatory factor and measurement invariance analyses of the Eating Disorder Examination Questionnaire in sexual minority men and women. Int J Eat Disord.

[35] Compte EJ, Nagata JM, Sepúlveda AR, Schweiger S, Sbdar LS, Silva BC (2019). Confirmatory factor analysis and measurement invariance of the eating disorders examination-questionnaire across four male samples in Argentina. Int J Eat Disord.

[36] Rand-Giovannetti D, Cicero DC, Mond JM, Latner JD (2020). Psychometric properties of the Eating Disorder Examination-Questionnaire (EDE-Q): a confirmatory factor analysis and assessment of measurement invariance by sex. Assessment.

[37] Scharmer C, Donahue JM, Heiss S, Anderson DA (2020). Factor structure of the Eating Disorder Examination—Questionnaire among heterosexual and sexual minority males. Eat Behav.

[38] World Health Organization. ICD-10: international statistical classification of diseases and related health problems: 10^th^ revision. 2^nd^ ed.; 2004.

[39] Hardt J (2008). The symptom checklist-27-plus (SCL-27-plus): a modern conceptualization of a traditional screening instrument. GMS Psycho-Soc-Med..

[40] Derogatis LR, Cleary PA (1977). Factorial invariance across gender for the primary symptom dimensions of the SCL-90. Br J Soc Clin Psychol.

[41] Hautzinger M, Bailer M, Worall H, Keller F (1994). Beck-Depressions-Inventar (BDI).

[42] Fairburn CG, Cooper Z, Fairburn CG, Wilson GT (1993). The eating disorder examination. Binge eating: nature, assessment and treatment.

[43] Hilbert A, Tuschen-Caffier B, Karwautz A, Niederhofer H, Munsch S (2007). Eating Disorder Examination-Questionnaire. Diagnostica.

[44] Hilbert A, Tuschen-Caffier B. Eating disorder examination. Verlag für Psychotherapie; 2006. p. 1–7.

[45] Hilbert A, Tuschen-Caffier B. Eating Disorder Examination. Vol. 2. Tübingen: dgvt-Verlag; 2016.

[46] Dziuban CD, Shirkey EC (1974). When is a correlation matrix appropriate for factor analysis? Some decision rules. Psychol Bull.

[47] Swami V, Barron D (2019). Translation and validation of body image instruments: challenges, good practice guidelines, and reporting recommendations for test adaptation. Body Image.

[48] Ferguson E, Cox T (1993). Exploratory factor analysis: a users’ guide. Int J Sel Assess.

[49] Horn JL (1965). A rationale and test for the number of factors in factor analysis. Psychometrica.

[50] Dinno A (2009). Exploring the sensitivity of Horn’s parallel analysis to the distributional form of random data. Multivariate Behav Res.

[51] Hayton JC, Allen DG, Scarpello V (2004). Factor retention decisions in exploratory factor analysis: a tutorial on parallel analysis. Organ Res Methods.

[52] Beavers AS, Lounsbury JW, Richards JK, Huck SW, Skolits GJ (2013). Practical considerations for using exploratory factor analysis in educational research. Pract Assess Res Eval.

[53] Howard MC (2016). A review of exploratory factor analysis decisions and overview of current practices: what we are doing and how can we improve?. Int J Hum Comput Interact.

[54] IBM Corporation. IBM SPSS Statistics for Windows 28.0. Armonk: International Business Machines Corporation; 2021.

[55] R Core Team. R: A Language and Environment for Statistical Computing. Vienna, Austria; 2022.

[56] Tierney N, Cook D (2023). Expanding tidy data principles to facilitate missing data exploration, visualization and assessment of imputations. J Stat Softw.

[57] O’Connor, B. P. Package “EFA.dimensions”—Exploratory Factor Analysis Functions for Assessing Dimensionality. 2023.

[58] Revelle W. psych: Procedures for psychological, psychometric, and personality research. Evanston, Illinois; 2022.

[59] Manukyan A, Çene E, Sedef A, Demir I (2014). Dandelion plot: a method for the visualization of R-mode exploratory factor analyses. Comput Stat.

[60] Zygmont C, Smith MR (2014). Robust factor analysis in the presence of normality violations, missing data, and outliers: empirical questions and possible solutions. Quant Method Psychol.

[61] Lim S, Jahng S (2019). Determining the number of factors using parallel analysis and its recent variants. Psychol Methods.

[62] Grilo CM, Reas DL, Hopwood CJ, Crosby RD (2015). Factor structure and construct validity of the Eating disorder Examination-Questionnaire in college students: further support for a modified brief version. Int J Eat Disord.

[63] Darcy AM, Hardy KK, Crosby RD, Lock J, Peebles R (2013). Factor structure of the Eating Disorder Examination Questionnaire (EDE-Q) in male and female college athletes. Body Image.

[64] Friborg O, Reas DL, Rosenvinge JH, Rø Ø (2013). Core pathology of eating disorders as measured by the Eating Disorder Examination Questionnaire (EDE-Q): the predictive role of a nested general (g) and primary factors. Int J Methods Psychiatr Res.

[65] Forsén Mantilla E, Birgegård A, Clinton D (2017). Factor analysis of the adolescent version of the Eating Disorders Examination Questionnaire (EDE-Q): results from Swedish general population and clinical samples. J Eat Disord.

[66] Bobko P, Schemmer FM (1984). Eigenvalue shrinkage in principal components based factor analysis. Appl Psychol Meas.

[67] Hair JF, Black WC, Babin BJ, Anderson RE (2010). Multivariate data analysis.

[68] Hilbert A, de Zwaan M, Braehler E (2012). How frequent are eating disturbances in the population? Norms of the Eating Disorder Examination-Questionnaire. PLoS One.

[69] Gideon N, Hawkes N, Mond J, Saunders R, Tchanturia K, Serpell L (2016). Development and psychometric validation of the EDE-QS, a 12 item short form of the Eating Disorder Examination Questionnaire (EDE-Q). PLoS ONE.

